# Scientific issues with rodent models of neuromyelitis optic spectrum disorders

**DOI:** 10.3389/fimmu.2024.1423107

**Published:** 2024-11-19

**Authors:** Yusen Huang, Tianwei Wang, Fangruyue Wang, Yujing Wu, Jia Ai, Ying Zhang, Meiyan Shao, Le Fang

**Affiliations:** ^1^ Department of Neurology, China-Japan Union Hospital of Jilin University, Changchun, China; ^2^ Department of Radiology, China-Japan Union Hospital of Jilin University, Changchun, China; ^3^ The Third Bethune Hospital of Jilin University, Changchun, China

**Keywords:** neuromyelitis optica spectrum disorders, autoimmune disorders, animal model, AQP4-IgG, autoantibodies

## Abstract

Neuromyelitis optica spectrum disorders (NMOSD) is a rare autoimmune disorder that causes severe inflammation in the central nervous system (CNS), primarily affecting the optic nerves, spinal cord, and brainstem. Aquaporin-4 immunoglobulin G antibodies (AQP4-IgG) are a diagnostic marker of the disease and play a significant role in its pathogenesis, though the exact mechanism is not yet fully understood. To develop rodent models that best simulate the *in vivo* pathological and physiological processes of NMOSD, researchers have been continuously exploring how to establish the ideal model. In this process, two key issues arise: 1) how the AQP4 antibody crosses the blood-brain barrier, and 2) the source of the AQP4 antibody. These two factors are critical for the successful development of rodent models of NMOSD. This paper reviews the current state of research on these two aspects.

## 1 Introduction

Neuromyelitis optica spectrum disorders (NMOSDs) are autoimmune central nervous system (CNS) disorders characterized by inflammation and demyelination of the optic nerves and spinal cord. They are characterized by a high relapse rate and a significant burden of disability, predominantly affecting young to middle-aged individuals. Despite extensive research, the pathogenic mechanisms underlying NMOSD remain elusive, highlighting a critical gap in current knowledge.

To effectively elucidate the pathogenesis of NMOSD, establishing a robust animal model is essential. In over 80% of NMOSD cases, autoantibodies against aquaporin-4 (AQP4) are detectable, serving as a key diagnostic marker. Once these antibodies penetrate the central nervous system, they bind to AQP4 on astrocytes, triggering a cascade of immune responses that result in the depletion of glial fibrillary acidic protein (GFAP) and a spectrum of pathological changes. These pathological changes include infiltration of macrophages and granulocytes, deposition of complement, demyelination, and axonal damage (1, 2). Rodents, particularly rats and mice, are frequently used as models for NMOSD research because of the similarity between the AQP4 protein sequences in humans and rodents (3). This similarity allows human AQP4-IgG to bind to rodent CNS AQP4, replicating the NMOSD pathology. However, challenges arise with the mouse model due to its weak and unstable complement system, which can inhibit the classical complement pathway. As a result, AQP4-IgG alone is often insufficient to activate the mouse’s complement system, requiring the additional injection of human complement to replicate NMOSD pathology (4, 5). In contrast, rats, with their functional complement system, require only the introduction of AQP4-IgG to develop NMOSD-like pathology.

During the investigation of model development, it was discovered that administering AQP4-IgG via peripheral routes (e.g., abdominally or intravenously) to mice or rats resulted in the positive AQP4-IgG serum level in these animals. Consequently, AQP4-IgG accumulates in peripheral organs that predominantly express AQP4, such as the trachea, stomach, kidneys, and skeletal muscle. Notably, AQP4-IgG deposition was absent in the central nervous system, except for the area postrema in the brain. Furthermore, no pathological changes were observed at the sites of deposition (6, 7). This finding is consistent with the observation in NMOSD patients, where AQP4-IgG can persist in peripheral blood for years without causing any symptoms (8). Further investigation (7) revealed that creating a small puncture in the brain of AQP4-IgG seropositive rats with a 28-gauge needle induced distinct NMOSD pathology around the needle track. This highlights a pivotal issue in developing NMOSD models: understanding how AQP4-IgG crosses the blood-brain barrier (BBB) to mimic the neuropathological processes observed in NMOSD patients.

Several research groups have attempted to develop NMOSD models in Lewis rats using the same experimental techniques. Although these efforts resulted in pathological changes similar to those in human NMOSD, discrepancies were observed in the resulting pathology (9–11). Further investigation revealed that AQP4-IgG in patients is polyclonal with varied affinities for AQP4 (9, 12). This elucidates the variability in pathological outcomes across identical experiments. During this period, antibodies such as rAb-53 (13) and E5415A (14) were selected for their higher affinity and their potential to induce more severe NMOSD pathology. This revelation highlights another pivotal issue in the NMOSD model development: the source of AQP4-IgG.

**Figure 1:**
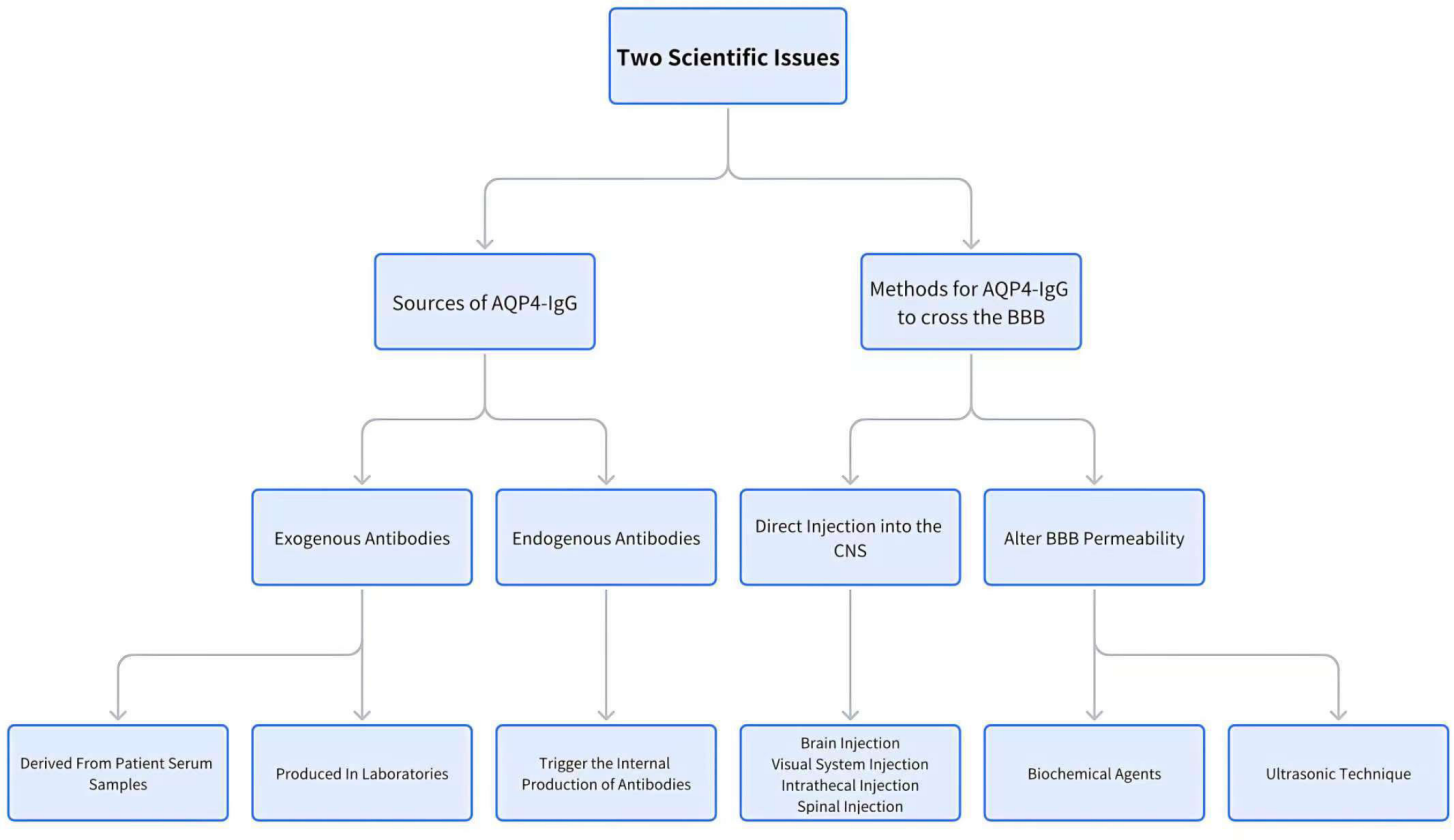
The discussion strategy along the manuscript.

Thus, understanding the mechanisms by which AQP4-IgG traverses the BBB and identifying the sources of AQP4-IgG are essential for the development of effective rodent models for NMOSD. This paper provides a comprehensive review of recent advancements and findings related to these key factors (**Figure 1**).

## 2 Methods for AQP4-IgG to cross the BBB

In animal model studies, researchers use two main methods to facilitate the transport of AQP4-IgG across the BBB. The first method involves directly injecting AQP4-IgG into the CNS, targeting specific regions such as the brain, optic nerve, and spinal cord. This method ensures precise delivery of AQP4-IgG to the targeted locations. The second method, known as the passive transfer method, focus on altering the permeability of the BBB to allow AQP4-IgG to enter the CNS. Based on the presence of serum AQP4-IgG positivity in experimental animals, this method modifies the BBB permeability to facilitate the entry of AQP4-IgG into the CNS. Both methods are designed to bypass the protective mechanisms of the BBB, facilitating the study of AQP4-IgG’s effects on the CNS (**Table 1**) (15).

**Table 1:** Rodent models of neuromyelitis optic spectrum diseases.

Methods to cross the BBB	Rodent models	Antibody source			Antibody dosage	Pathological alterations							Behavioral phenotype	Pain phenotype	Time point (s) examined	Reference
		Patient serum: Extraction or Direct use	Monoclonal antibody	Autoimmune		Sites of pathology	AQP4 loss	GFAP loss	Infiltration cell type(s)	Complement deposition	Myelin loss	Axonal injury				
Needle injury	CD1 mice	Yes			28 μL or 16.8 μL	Brain	Yes	Yes	Macrophages	Yes	Yes	Yes	Turn right	ND	12h-7d	Saadoun et al (16)
	C57BL/6 mice	Yes			6 μl	Brainstem, Cerebellum, Periventricular region	Yes	Yes	Monocytes	Yes	Yes	ND	ND	ND	7d	Asgari et al (26)
	OFA rats	Yes			400 μg	Brain, Spinal cord, Optic nerve	Yes	No	Mild inflammation (type not specified)	No	Yes	Yes	Impaired motor behavior	ND	7d	Marignier et al (27)
	SD rats	Yes			2μl	Optic nerve, Retina	Yes	Yes	CD11+ cells	ND	ND	Yes	ND	ND	7-14d	Matsumoto et al (21)
	SD rats	Yes			2μl	Optic nerve, Retina	Yes	Yes	ND	ND	ND	ND	ND	ND	21d	Nobuyoshi et al (22)
	SD rats	Yes			6μl	Optic nerve, Retina	No	No	Microglia, Macrophages	ND	Yes	Yes	ND	ND	21d	Zhang et al (23)
	Lewis rats		Yes		20μg	Spinal cord	Yes	Yes	ND	ND	ND	ND	ND	Mechanical pain thresholds are reduced	1-28d	Ishikura et al (28)
	Lewis rats		Yes		10μg	Brain	Yes	Yes	Neutrophils, Macrophages	Yes	Yes	No	ND	ND	5d	Asavapanumas et al (13)
	CD1 mice		Yes		Retrobulbar 1μg, intravitreal 1or 3μg, near the optic chiasm (Single):5μg, near the optic chiasm (Continuous):3.3μg×3d	Optic nerve	Yes	Yes	Neutrophils, Macrophages, Microglia	Yes	Yes	ND	ND	ND	3d	Asavapanumas et al (19)
	SD rats		Yes		40 μg	Retina	Yes	No	Microglia	Yes	ND	ND	ND	ND	6 h- 30 d	Felix et al (20)
	CD59-/- mice		Yes		10 μg	Spinal cord	Yes	Yes	Neutrophils, Macrophages/Microglia	Yes	Yes	ND	Hind limb weakness	ND	2d	Zhang et al (25)
	Lewis rats		Yes		2mg	Brain	Yes	Yes	Neutrophils, Macrophages	Yes	Yes	ND	ND	ND	5d	Asavapanumas et al (7)
	Lewis rats		Yes		1.5 μL	Optic nerve	Yes	Yes	Macrophages, Neutrophils	ND	Yes	Yes	The pupillary light reflex was severely impaired	ND	2d, 4d, 7d and 24d	Morita et al (53)
EAE	Lewis rats	Yes			1mg	Spinal cord	Yes	Yes	T cells, Macrophages	Yes	Yes	Yes	ND	ND	24h	Bradl et al (10)
	Lewis rats	Yes			20 mg×4d	Spinal cord	Yes	Yes	Macrophages, Neutrophils	Yes	ND	ND	Clinical score increase	ND	4d	Kinoshita et al (11)
	C57/BL6 mice	Yes			10 mg	Optic nerve, Spinal cord	Yes	No	Few granulocytes	ND	Yes	ND	EAE score increasing	ND	19d, 62d	Saini et al (30)
	Lewis rats		Yes		2.5 mg	Spinal cord	ND	Yes	No	Yes	ND	Yes	ND	ND	30h	Bennett et al (9)
	Lewis rats		Yes		0.01mg-1mg	Brainstem, Optic chiasm, Spinal cord	Yes	Yes	Neutrophils	Yes	Yes	Yes	Clinical score increase	ND	2d	Kurosawa et al (14)
	Lewis rats		Yes		1, 3 or 6 mg/kg	Spinal cord	Yes	Yes	Neutrophils, Macrophages	ND	ND	ND	Assessment of Clinical Disability	Paw withdrawal thresholds significantly decrease	22d	Iwamoto et al (31)
	C57BL/6 mice	ND	ND	ND	ND	Optic nerve	ND	ND	ND	ND	ND	ND	EAE score increasing	ND	14-60d	Guo et al (41)
Ultrasonic Technique	C57BL/6 WT mice	Yes			100µg	Optic nerve, Spinal cord	Yes	Yes	Monocytes predominate	Yes	Yes	ND	ND	ND	24h	Luo et al (38)
	C57BL/6 WT mice	Yes			100 μL	Optic nerve, Spinal cord	Yes	Yes	Neutrophils	ND	Yes	ND	EAE score increasing	ND	24d	Xiang et al (46)
	SD rats		Yes		5mg/kg	Brain, Spinal cord	Yes	Yes	Microglia, CD45+ cells	Yes	Yes	ND	ND	ND	5d	Yao et al (37)
CFA and PTx	C57BL/6 mice	Yes			4.0 mg×8d	Spinal cord	Yes	Yes	Microglia, Macrophages, Neutrophils	No	Yes	Yes	Motor weakness	ND	15d	Yick et al (32)
	C57BL/6N mice			Yes	ND	Spinal cord	Yes	Yes	Neutrophils, eosinophils, Microglia, Few Macrophages	ND	Yes	Yes	Motor disorders	ND	42d	Yick et al (56)
	C57BL/6J mice			Yes	ND	Spinal cord	Yes	Yes	Yes (type unspecified)	Yes	ND	ND	Assessment of clinical signs	ND	4w	Serizawa et al (57)
AQP4-specific T cells	Lewis rats	Yes			10mg	Brain, Spinal cord	Yes	ND	Macrophages, T cells	Yes	No	ND	ND	ND	24h	Pohl et al (34)
	Lewis rats	Yes			10mg	Brain, Spinal cord	Yes	Yes	Macrophages, T cells	Yes	ND	ND	Partial loss of tail tonus	ND	7d	Zeka et al (35)
	Lewis rats	Yes			Yes	Optic nerve, Retina	Yes	Yes	T cells, Macrophages	Yes	Yes	Yes	ND	ND	5d	Zeka et al (45)
	C57BL/6 mice	ND	ND	ND	ND	Optic nerve, Retina, Spinal cord	ND	Yes	T cells	ND	No	Yes	Paralysis	ND	2d	Sagan et al (44)
Undamaged	CD1 mice		Yes		20μg	Area postrema	Yes	Yes	ND	ND	Yes	ND	ND	ND	1h, 24h	Ratelade et al (6)
	Lewis rats		Yes		1mg×3d	Brain, Spinal cord	Yes	Yes	Macrophages	Yes	No	No	ND	ND	24h, 48h, 120h	Hillebrand et al (54)
Intrastriatal injection of interleukin-1 beta	Lewis rats	Yes			10mg or 5mg	Brain	Yes	ND	Neutrophils	Yes	No	ND	ND	ND	18-24h	Kitic et al (36)

ND, not reported.

### 2.1 Direct injection of AQP4-IgG into the CNS

Administering a minimal amount of AQP4-IgG directly into the CNS effectively induces NMOSD pathology in experimental animals, allowing for the precise quantification of demyelination behavior (5). This method has become a preferred strategy for replicating NMOSD, as the pathological changes observed in the generated NMOSD models closely resemble those seen in humans, providing a strong basis for further NMOSD research (7). However, it’s essential to acknowledge that lesions in NMOSD patients typically occur around blood vessels (2), which is not entirely replicated in the animal models, in which lesions primarily localize at the injection sites. Additionally, needle-induced damage and subsequent inflammatory responses may obscure the differences between experimentally induced lesions and the natural pathology of NMOSD, complicating the interpretation of results.

#### 2.1.1 AQP4-IgG brain injection

**Figure 2:**
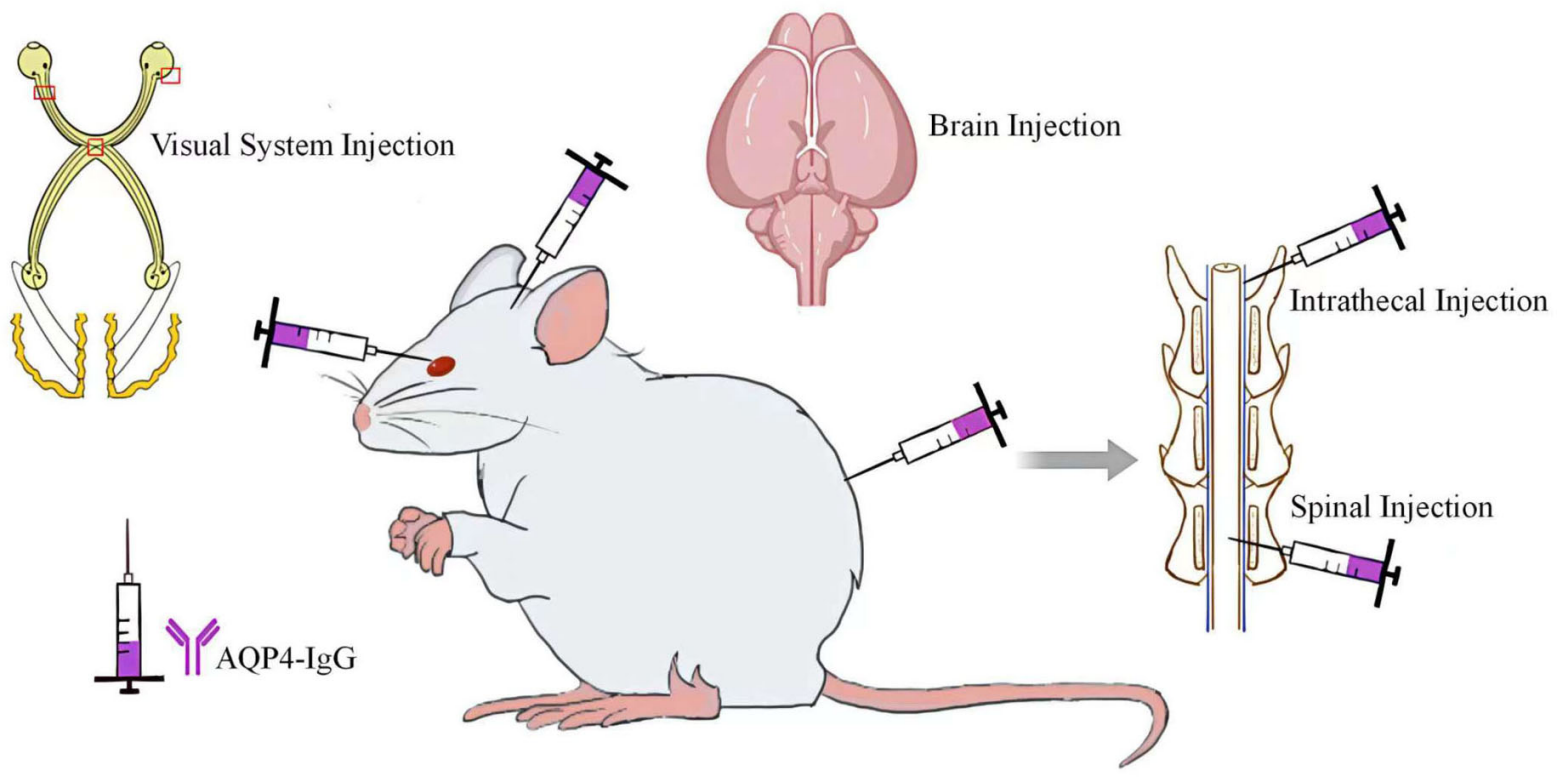
Direct Injection of AQP4-IgG into the CNS.

Saadoun et al. (16) introduced AQP4 into the mouse brain, resulting in a mild inflammatory response at the injection site without evidence of AQP4 or GFAP deletion, demyelination, or complement activation. Further studies using Chinese hamster ovary (CHO-AQP4) cells, which stably expressing AQP4 revealed that these cells underwent complement deposition and lysis in about half of the cases when exposed to AQP4-IgG and human complement. In contrast, AQP4-IgG combined with mouse complement did not lead to cell lysis. Further research involving the injection of AQP4-IgG and human complement into the mouse brain caused NMOSD-like pathological changes (**Figure 2**), including the reduction of AQP4 and GFAP, inflammatory cells infiltration, demyelination, and accumulation of activated complement components around blood vessels. These findings were supported by subsequent experiments showing that complement inhibitors significantly reduced the inflammation induced by AQP4-IgG and human complement, highlighting the critical role of the complement pathway in NMOSD pathology. Building on the method developed in mice, Asavapanumas et al. (13) developed a rat model that mimics NMOSD pathology without the need for human complement. In this experiment, the importance of the complement system in NMOSD was further demonstrated by a significant reduction in lesion size after complement inactivation with cobra venom factor.

#### 2.1.2 AQP4-IgG visual system injection

A significant proportion of patients suffer from chronic visual impairment, with a specific group experiencing recurrent episodes of optic neuritis, that can lead to permanent blindness (17, 18). Given the prevalence and significant disability caused by optic neuritis in NMOSD patients, in-depth research into its pathogenesis and the development of stable models for study are essential.

Asavapanumas (19) and colleagues explored a variety of approaches to deliver AQP4-IgG and complement to the visual system of mice, including retrobulbar infusion, intravitreal injection, a singular intracranial injection near the optic chiasm, and continuous intracranial infusions spanning three days (**Figure 2**). However, only the three-day continuous intracranial infusion approach near the optic chiasm was able to induce NMOSD-like pathological changes.

To investigate NMOSD-like pathological changes in the retina, AQP4-IgG was administered intravitreally. Asavapanumas (19) injected either 1 or 3μg AQP4-IgG along with 0.5 μL of human complement into the vitreous body of CD1 mice. This led to the observation of AQP4-IgG binding to AQP4 on the retinal Müller cells, but no retinal lesions were identified. In contrast, Felix et al. (20) used a similar approach, administering 40μg of AQP4-IgG (utilizing the high-affinity antibody rAb-53 as well) into the vitreous body of SD rats. They reported loss of AQP4 in Müller cell, elevated GFAP indicating a glial response, microglial activation, minimal leukocyte infiltration, and a slight reduction in retinal ganglion cells (RGCs) along with thinning of the ganglion cell complex (GCC). The differing outcomes of these studies highlight the need for further research to determine if the variance is due to antibody dosage, potential complement system deficiencies in the mice, or genetic differences.

Separately, Matsumoto et al. (21) Nobuyoshi et al. (22) and Zhang et al. (23) found a decrease in AQP4 and GFAP, a reduced density of RGCs, and significant infiltration of inflammatory cells after exposing the optic nerve via blunt dissection and injecting AQP4-IgG under the optic nerve sheath. This model reliably induces optic neuritis and has been used to evaluate the protective effects of medications on the optic nerve in NMOSD patients, thereby enhancing our understanding of mechanisms of NMOSD.

#### 2.1.3 AQP4-IgG intrathecal injection

In patients diagnosed with NMOSD, in addition to optic neuritis, longitudinal extensive transverse myelitis stands out as another notable characteristic (24). To replicate the spinal cord alterations observed in NMOSD patients, researchers have undertaken various studies, with intrathecal injection of AQP4-IgG being the most prevalent method employed (**Figure 2**). Zhang et al. (25) administered a single intrathecal injection of AQP4-IgG and human complement between the L5 and L6 levels in both wild-type and CD59-deficient mice. Their findings indicated that CD59-deficient mice developed longitudinally extensive white matter lesions with AQP4 and GFAP loss, astrocyte activation, C5b-9 deposition, granulocyte infiltration, and demyelination, whereas wild-type mice displayed no significant abnormalities. CD59 serves as a crucial complement regulatory protein in human astrocytes and this divergent outcome suggests a protective function of CD59 in this model. Furthermore, the investigation revealed that lesions were predominantly manifested in the lumbar spinal cord of CD59-deficient mice, potentially due to the restricted diffusion of AQP4-IgG and/or human complement. Previous study (26) has illustrated that a singular intrathecal injection of AQP4-IgG combined with complement infusion into the cisterna magna of mice can elicit NMOSD-like pathology in regions such as the cerebellum, brainstem, periventricular areas, and around the fourth ventricle. However, no notable changes were observed in the spinal cord, confirming the above findings.

Marignier et al. (27) employed osmotic mini-pumps to continuously infuse AQP4-IgG into the cerebral ventricles of rats for a duration of 7 days. Following this procedure, human IgG was detected in the brains, spinal cords, and optic nerves of the rats. These areas showed evidence of AQP4 loss and the presence of reactive astrocytes, particularly notable in the spinal cord and optic nerves. Additionally, demyelination and axonal damage were observed in the spinal cord. This method, similar to intrathecal injection, allows the infusion of AQP4-IgG directly into the cerebrospinal fluid, thus simulating NMOSD-like pathological changes in experimental animals.

#### 2.1.4 AQP4-IgG spinal injection

Compared to the intrathecal injection method, the principal drawback of spinal cord injection is the trauma to the spinal cord. Ishikura et al. (28) conducted a study in which they performed a laminectomy at the thoracic level (Th10) in rats and injected either AQP4-IgG or control human IgG into the exposed spinal cord (**Figure 2**). Despite the inevitable puncture damage and pathological changes caused by antibody introduction at the injection site, a broader range of lesions was observed in certain histological sections of the AQP4-IgG-treated group, accompanied by astrocyte proliferation at the periphery of the injury. Furthermore, this model effectively mimics the acute pain process in patients with NMOSD. The study found that AQP4-IgG induces the release of ATP from astrocytes, and the application of ATP receptor antagonists can reverse mechanical allodynia in with AQP4-IgG-treated rats, thereby highlighting the central role of ATP in the pain mechanism associated with NMOSD. Although this model successfully replicates the acute phase of pain in NMOSD patients, it falls short in simulating NMOSD pathological changes within the spinal cord. In addition, as pain in NMO patients becomes more pronounced during the chronic phase (29), this model exhibits certain limitations.

### 2.2 AQP4-IgG passive transfer method for altering BBB permeability

**Figure 3:**
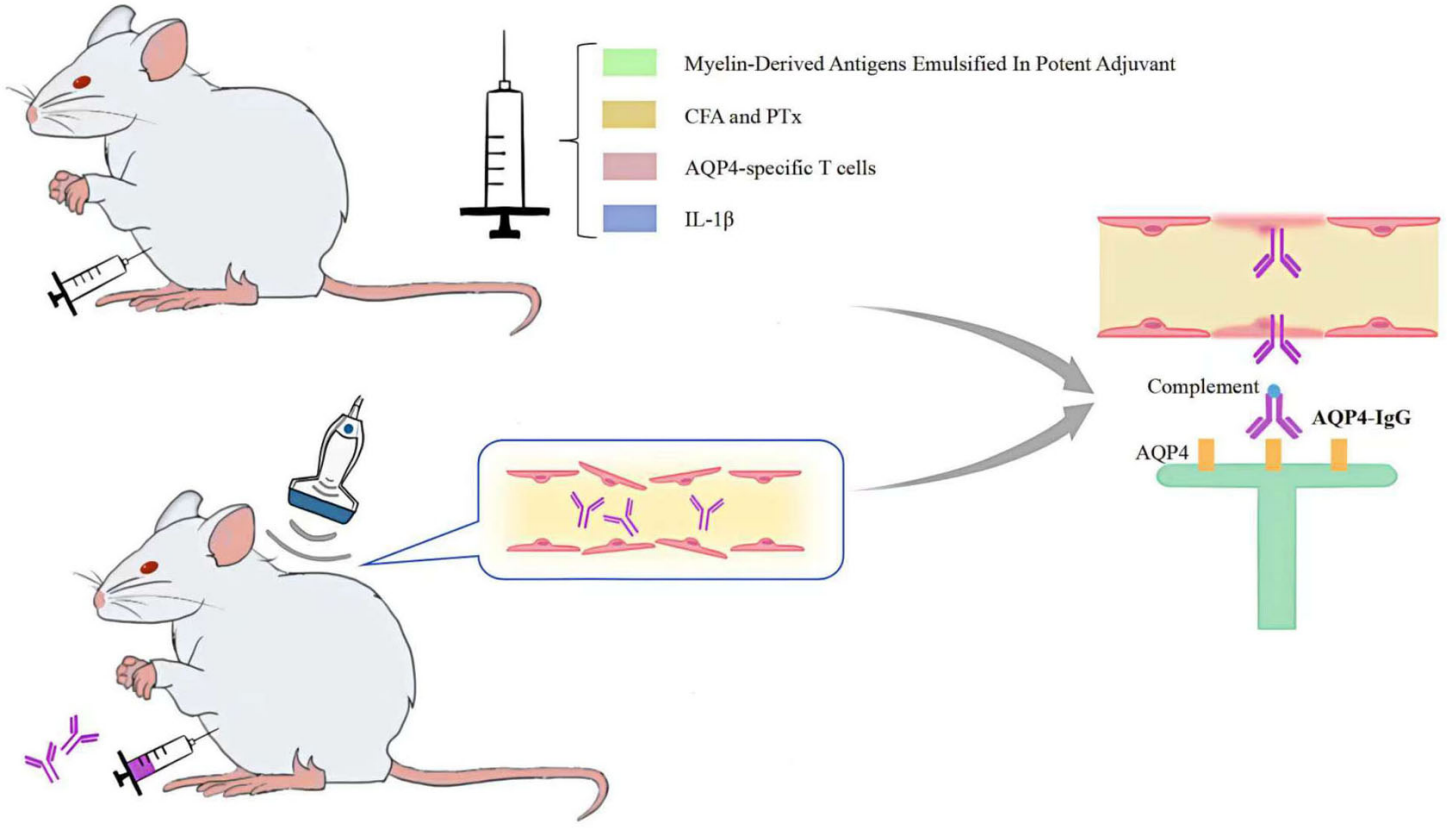
Methods for altering BBB permeability.

The AQP4-IgG passive transfer method of altering BBB permeability more accurately reflects the human physiological context than the NMOSD model, which is generated by direct injection of AQP4-IgG into the CNS. On the basis of seropositive AQP4-IgG in experimental animals, peripheral injection of myelin-derived antigens emulsified in potent adjuvants (30, 31), administration of complete Freund’s adjuvant (CFA) and pertussis toxin (PTx) (32, 33), peripheral injection of AQP4-specific T cells (34, 35) injection of IL-1β into the striatum (36) pulsed focused ultrasound with microbubbles (37), or microbubble-enhanced low-frequency ultrasound (38) can all induce changes in BBB permeability, thereby facilitating the entry of AQP4-IgG across the BBB into the central nervous system to produce NMOSD pathological changes (**Figure 3**).

#### 2.2.1 Myelin-derived antigens emulsified in potent adjuvant

Peripheral injection of myelin-derived antigens emulsified in potent adjuvants is a prevalent induction method for experimental autoimmune encephalomyelitis (EAE). These antigens are typically emulsified in Complete Freund’s Adjuvant (CFA), which contains components of mycobacterial membranes and/or pertussis toxin (PTx). This formulation facilitates the induction of an inflammatory response in the CNS of animal models, leading to alterations in the permeability of the blood-brain barrier (39, 40). Subsequent peripheral injection of AQP4-IgG exacerbates EAE and induces NMOSD-like pathological alterations in the CNS (11, 41). The EAE model has been pivotal in the initial exploration of the pathomechanisms underlying NMOSD, especially in validating the pathogenicity of AQP4-IgG (10, 11). However, current research indicates that the pathogenesis of NMOSD is largely attributable to humoral immune responses against AQP4 (42), whereas EAE models the targeted response of myelin-sensitized T cells (40), Furthermore, the EAE-induced changes in blood-brain barrier permeability and the resultant inflammatory response in the CNS may confound the distinct inflammatory processes inherent to NMOSD.

#### 2.2.2 CFA and PTx

The study by Chan et al. (33) reveals that the use of CFA without myelin-derived antigens, containing inactivated H37Ra MTB, in combination with PTx, disrupts the BBB. This disruption allows AQP4-IgG to enter the CNS. When the BBB is breached in mice using this method, administering AQP4-IgG intraperitoneally (2mg per dose over three days) leads only to the asymptomatic loss of AQP4 and astrocyte activation in the spinal cord. This result supports the important role of complement activation in the astrocytic damage seen in NMOSD, which includes inflammatory demyelination. However, a separate experiment (32), showed that after BBB disruption, administration of a higher dose of AQP4-IgG (4mg per dose over eight days) caused more typical NMOSD changes in the spinal cord. This suggests that a higher dose of AQP4-IgG can trigger astrocytic toxicity without complement activation, probably through antibody-dependent cell-mediated cytotoxicity (ADCC), mainly involving macrophages and microglia, with minimal neutrophil involvement. Such a methodology allows for the induction of NMO pathological changes in experimental animals in the absence of myelin-derived antigen-specific T cells.

#### 2.2.3 AQP4-specific T cells

Currently, there is a paucity of understanding regarding the involvement of AQP4 specific T cells in the humoral and cellular immunity of the CNS (43). Previous studies have endeavored to generate mammalian NMOSD models by the peripheral injection of AQP4-specific T cells. However, the majority of these models are considered unsuccessful: while some models exhibited extensive inflammation and demyelination, the quintessential hallmark of NMOSD-AQP4-loss was not observed. This may be due to the relatively weak encephalitogenic potential of AQP4-specific T cells, which localize predominantly to the meninges and therefore infiltrate the CNS parenchyma to a minimal extent (35). Nevertheless, the peripheral co-injection of AQP4-IgG, in conjunction with the presence of AQP4-specific T cells, resulted in NMOSD pathological changes within the CNS, including AQP4 loss (34, 35, 44). This suggests that AQP4-specific T cells may alter the permeability of the blood-brain barrier, thereby facilitating the entry of AQP4-IgG into the CNS. Intriguingly, the study by Zeka et al. (45) demonstrated that utilizing T-cell immunization with specific AQP4 peptides could generate AQP4_268–285_-specific T cells. Intraperitoneal injection of these cells alone induced T-cell infiltration into the retina, leading to the opening of the BRB, although insufficient to induce retinitis in all experimental animals. However, the combined intraperitoneal injection of AQP4_268–285_-specific T cells and AQP4-IgG induced retinitis more consistently and resulted in the loss of AQP4 in Müller cells within the retina. Such models have contributed to understanding the impact of AQP4-specific T cells on the CNS in NMOSD patients, but they overlook the pivotal role of humoral immunity and complement activation in NMOSD, and the NMOSD pathological changes produced are not ideal.

#### 2.2.4 IL-1β

In an effort to elucidate the effects of cytokines generated during neuroinflammatory processes on the BBB permeability, researchers (36) introduced AQP4-IgG or a control human IgG into rats peripherally. Subsequently, administration of IL-1β, TNF-α, IFN-γ, and CXCL2 into the striatum was observed to result in extensive seepage of human IgG into the substance of the central nervous system. Notably, only IL-1β was responsible for inducing NMOSD-like pathological changes at sites remote from the injection sites. The pathological changes are localized to a diminutive region adjacent to the injection site, confined exclusively to the cerebral hemisphere correlating with the injection side, and show a smaller extent of lesions compared to those observed in humans. Moreover, there is a considerable variability in both the frequency and quantity of lesions, which can range from one to eight. Despite these limitations, this model is proving to be a useful tool for studying the pathological impacts of inflammatory cytokines in AQP4-IgG-positive animals on the central nervous system and providing a basis for therapeutic strategies to target these cytokines.

#### 2.2.5 Ultrasonic technique

With the continuous advancement of ultrasonography, Guan et al. (38) utilized microbubble-enhanced low-frequency ultrasound to reversibly open the BBB in the EAE model. Following this, they peripherally injected AQP4-IgG and complement into mice, facilitating the molecules’ entry into the CNS. This innovative method required only a minimal amount of AQP4-IgG to induce the characteristic pathological changes associated with NMOSD in the thoracic spine and optic nerve, compared to previous EAE models combined with AQP4-IgG. However, within the brain tissue, the observation was limited to the loss of GFAP and AQP4, with no evidence of inflammation or demyelination, a phenomenon that may be related to species variation in AQP4-IgG. Building on this experiment, Xiang et al. (46) integrated several control groups into their research, solidifying the evidence that a minimal quantity of AQP4-IgG can lead to severe pathology in both the spinal cord and optic nerve. Based on AQP4-IgG seropositivity in rats, Yao et al. (37) demonstrated that characteristic NMOSD pathological changes could be induced with pulsed focused ultrasound and microbubble treatment of the BBB, importantly, without causing potential inflammation or damage to the rats. A recent study (47) discovered that microbubble-assisted low-intensity pulsed ultrasound, optimized at an acoustic pressure of 0.15 MPa for 60 seconds, can transiently open the BRB in mouse eyes. This method allows drugs up to 150 kDa to penetrate, increasing the concentration of drug in the retina without causing any discernible adverse effects. Given that the molecular size of AQP4-IgG is approximately 150 kDa, which falls within the permissible range for BRB permeation observed in this study, this method holds potential for the development of NMOSD retina-related models in the future. Ultrasound technology, with its ability to efficiently, instantaneously, and safely open the BBB and BRB without inducing additional potential damage, shows great promise for application in NMOSD models. However, to fully harness this technology, further research is required to identify the optimal ultrasound exposure parameters, including acoustic pressure, frequency, ultrasound power, burst repetition frequency, and burst length. In addition, factors beyond the ultrasound device, such as tissue vascular density and microbubble characteristics (size, concentration distribution, and circulation time), also play a crucial role in influencing the extent of BBB or BRB opening (48).

## 3 AQP4-IgG source

**Figure 4:**
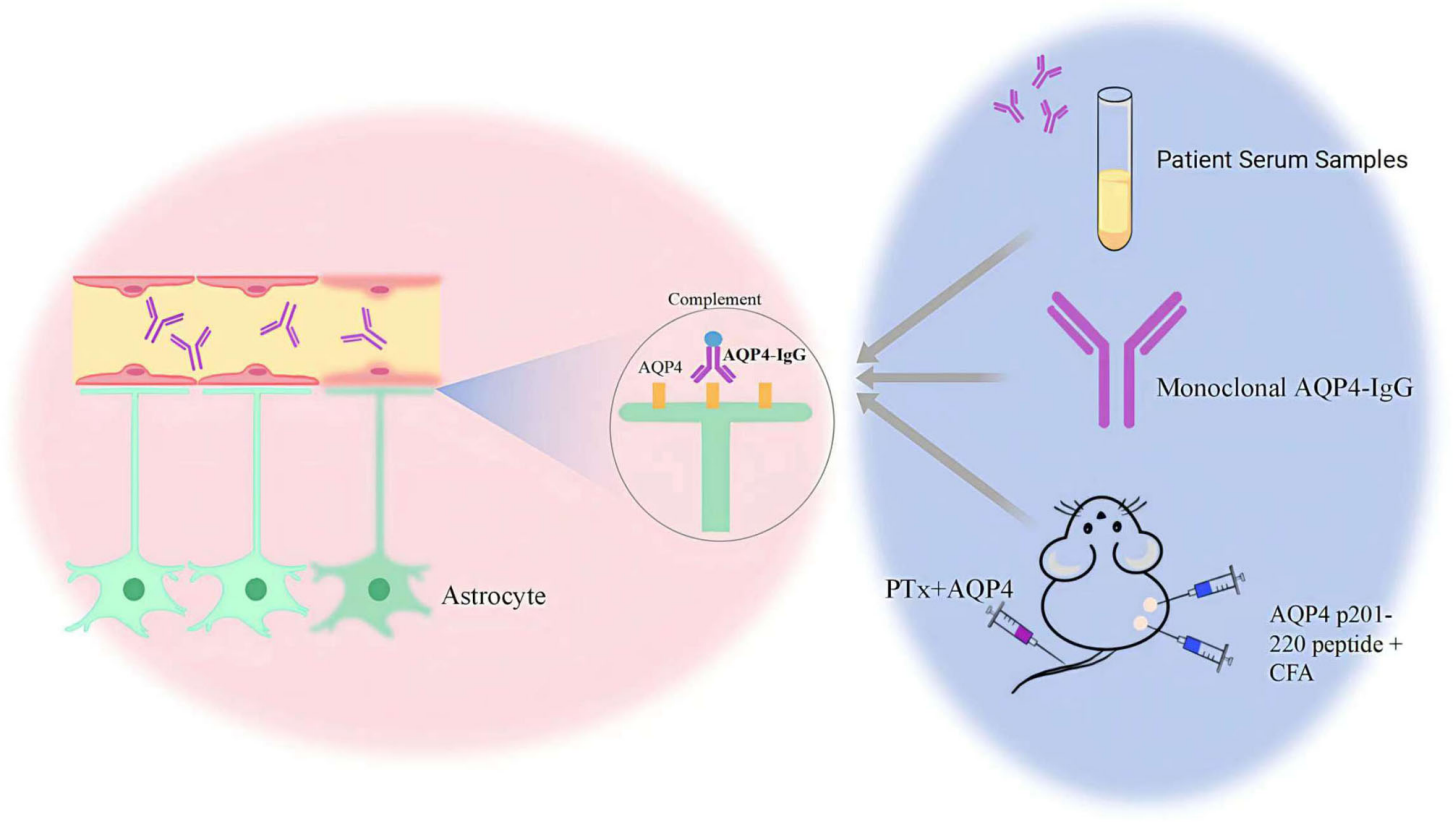
The sources of AQP4 IgG includes exogenous and endogenous antibodies.

It is unknown how AQP4-IgG is produced naturally in humans or what conditions promote its production. Wilson found that naïve B cells from NMOSD patients could produce AQP4-IgG *in vitro* in the absence of antigen after differentiation into antibody-secreting cells (49). Subsequent studies have similarly demonstrated this and suggested that autoreactive naïve B cells resulting from early B-cell tolerance checkpoint defects in NMOSD patients may contribute to the production of pathogenic anti-AQP4 autoantibodies (50). The B cell checkpoint eliminates B cells expressing the autoreactive B cell receptor on the way to B cell development, thus preventing autoantigen recognition and autopeptide presentation to T cells. Interestingly, defects in the B cell tolerance checkpoint are associated with many autoimmune diseases such as systemic lupus erythematosus (SLE), desiccation syndrome (SS), myasthenia gravis (MG) and others (51, 52), and a high proportion of coexisting autoimmune diseases and antibodies are often present in patients with NMOSD. The AQP4-IgG used in current research can be divided into two main types: exogenous and endogenous antibodies. Exogenous antibodies, including those isolated and purified from the serum of NMOSD patients and lab-generated monoclonal AQP4-IgG, are primarily used in animal models to study NMOSD. Recent advances in experimental techniques have led researchers to explore the potential of inducing endogenous AQP4-IgG production. This is achieved by triggering the autoimmune process in experimental animals, thereby generating AQP4-IgG to mimic the pathological process of NMOSD more closely (**Figure 4**).

### 3.1 Exogenous antibody

#### 3.1.1 AQP4-IgG derived from patient serum samples

AQP4-IgG isolated and purified from patient serum samples is widely used in the development of NMOSD models due to its wide availability and the simplicity of antibody extraction methods. Notably, AQP4-IgG present in the serum of NMOSD patients is polyclonal (9, 12) and exhibits considerable variability in pathogenicity and affinity among patients (53). Additionally, the affinity of AQP4-IgG for AQP4 across different species may vary between species, potentially leading to attenuated NMOSD pathological changes and functional impairments when using AQP4-IgG from different species. This heterogeneity underscores the possibility of disparate outcomes in identical experimental setups (54). Moreover, AQP4-IgG purified from patient serum frequently contains a mix of other IgGs and soluble factors, which may affect the research on AQP4-IgG (55).

#### 3.1.2 Monoclonal AQP4-IgG produced in laboratories

Monoclonal AQP4-IgG antibodies, synthesized in laboratories, are specifically chosen for their high affinity during the screening process. This selection results in a more efficient binding to the AQP4 antigen compared to polyclonal AQP4-IgG derived from patient serum, enabling the use of fewer antibodies to trigger NMOSD-like pathological changes (13). These changes have the potential to be more severe and widespread (14). A study by Hillebrand et al. (54) underscores this efficiency, demonstrating that administering a high-affinity monoclonal antibody, E5415A, via intraperitoneal injection in rats, led to extensive NMOSD pathological changes and clinical symptoms analogous to those observed in NMOSD patients. However, the production of AQP4-IgG monoclonal antibodies is challenging due to the complexity of their manufacture and high costs, limiting their widespread use.

Exogenous AQP4-IgG, which is essentially of human origin, acts as a foreign antibody when introduced into experimental animals. This introduction is capable of inducing NMOSD pathological changes in these animals, yet it fails to replicate the immune response elicited by the body’s own AQP4 antibodies production.

### 3.2 Endogenous antibodies

After developing models utilizing external antibodies, researchers have shifted their focus to triggering the internal production of antibodies in lab animals to delve deeper into the mechanisms and pathology of NMOSD. In a notable study, Yick et al. (56) conditioned mice with CFA and PTx before administering plasmids carrying the M23 variant of the mouse AQP4 protein into their anterior tibial muscles through electroporation on predetermined schedules. The electroporation not only facilitated the plasmid delivery but also caused muscle damage and inflammation, thereby sparking AQP4-specific immune responses. This approach led to the detection of AQP4 autoantibodies in the mice’s blood, triggering an autoimmune reaction against the naturally occurring AQP4. Another investigation (57) demonstrated that when the AQP4 p201-220 peptide was emulsified with CFA and used for multipoint subdermal immunization of mice, along with the peripheral administration of Pertussis Toxin, it effectively induced the production of AQP4 autoantibodies. This method not only generated AQP4 autoantibodies but also led to the development of clinical symptoms and pathological changes characteristic of NMOSD, with a high success rate in the model.

These studies underline the effectiveness of inducing AQP4-IgG through innate immune reactions in animal models, offering an accurate simulation of NMOSD’s pathogenesis in humans. This method opens up new pathways for understanding the disease and developing potential treatments.

## 4 Prospect

Currently, models of mammals are primarily focused on the disease’s acute phase. However, the recurrent nature of NMOSD, evidenced by the relapses occurring in almost all untreated patients (58), highlights the necessity for models that encompass both the remission and relapse phases for effective long-term management. This underscores an urgent need for models capable of simulating the remission phase to explore the mechanisms of recurrence further. Prior research indicates that creating a model capable of simulating the long-term remission phase in NMOSD requires administering repeated doses of AQP4-IgG to the experimental subjects. However, this approach risks inducing serum sickness due to the repeated introduction of foreign antibodies, characterized as a classic Type III hypersensitivity reaction (59). The advent of endogenous antibody models, however, offers a promising solution by circumventing the issue of serum sickness, thereby paving the way for the development of more comprehensive NMOSD models.

In patients with NMOSD, the prevalence of chronic pain is approximately 72% to 86% (60), with neuropathic pain being the most common manifestation (61). Research conducted by Ayzenberg et al. (62) discovered that over one-third of patients suffering from chronic pain also concurrently experience depression, with 51.5% of these individuals afflicted by moderate to severe depression. This study elucidates a close correlation between the intensity of pain, particularly neuropathic pain, and the incidence of depression. It also highlights that, despite symptomatic treatment, the majority of patients continue to endure moderate or severe pain. Consequently, investigating methodologies to alleviate chronic pain, especially during remission periods, is imperative for enhancing patient quality of life. The current understanding of NMOSD-related pain mechanisms remains limited. Although some studies have confirmed the presence of neuropathic pain in mammalian models of NMOSD, most of these studies have only considered pain as a complication of NMOSD. In addition, there are fewer pain comorbidity studies for this model, as well as a lack of well-established animal models of NMOSD pain. Studies have shown that there is a correlation between AQP4 and neuropathic pain (63). It has been shown that AQP4-IgG binding to AQP4 leads to an increase in extracellular glutamate. And excessive accumulation of glutamate causes abnormal neuronal excitation leading to pain (64). In addition, AQP4-IgG can mediate the extracellular release of ATP from astrocytes (28), and the released ATP may activate the P2X3 receptor, which may increase the pronociceptive effects of astroglial activation in NMOSD (adds to the pronociceptive effects of astroglial activation in NMOSD) (29) and other findings all suggest that the AQP4-IgG-based mammalian model of NMOSD may be an effective tool for studying pain in NMOSD. Given the ubiquity and severity of chronic pain, specifically neuropathic pain, in NMOSD patients, future animal model experiments should incorporate more comprehensive evaluations of chronic pain and investigations into its mechanisms. This approach will facilitate a better understanding of its neuropathological impact and the interplay with mood disorders, such as depression. Moreover, considering the persistent nature of chronic pain during the remission phases of NMOSD patients, the development of models that simulate the human condition of chronic pain during the chronic phase of NMOSD holds significant potential for providing more holistic and personalized treatment strategies. This approach will enhance our comprehension of its neuropathological effects and the interaction with mood disorders, including depression. Furthermore, given the persistent nature of chronic pain even during the remission phases of NMOSD patients, developing models that accurately reflect the human experience of chronic pain in NMOSD’s chronic phase is crucial. Given the enduring nature of chronic pain even during the remission periods in individuals afflicted with NMOSD, it is crucial to engineer a chronic phase model that mirrors the human experience of chronic pain within this context. Such a model is indispensable for the formulation of treatment strategies that are not only comprehensive but also tailored to the individual needs of the patient.

Research indicates that NMOSD, when positive for AQP4-IgG, is not only associated with a wide range of autoantibodies, including those extractable nuclear antigen (ENA), antinuclear antibodies (ANA), acetylcholine receptor muscle (mAChR), double-stranded DNA (dsDNA), and ganglionic acetylcholine receptor (gAChR), but also linked to several autoimmune diseases such as systemic lupus erythematous (SLE), Sjogren’s syndrome (SS), antiphospholipid syndrome (APS), and myasthenia gravis (MG) (65). This wide array of associations suggests that the pathogenesis of NMOSD may involve intricate immunoregulatory and genetic factors. However, current NMOSD animal models are mainly focused on the AQP4-IgG antibody, neglecting the potential influence of other autoimmune diseases and antibodies on the disease’s onset and progression. Therefore, broadening the scope of NMOSD animal model research to include these factors could represent a novel direction for investigation. Despite the high prevalence of concomitant autoimmune diseases and antibodies among NMOSD patients, studies often isolate these factors, which may overlook their collective impact on the disease. For instance, a retrospective study examining 16,360 NMOSD hospital cases revealed that approximately 8.71% (1,425 cases) were also diagnosed with SLE or SS (66). Additionally, in a case-control study with 117 NMOSD patients, about 2% simultaneously suffered from MG, and 11% tested positive for AChR (67). These findings suggest a complex interaction between NMOSD and other autoimmune diseases. Consequently, although research using animal models to study these co-occurring autoimmune diseases and autoantibodies in NMOSD might require significant resources and impact a smaller population, it is crucial. This research holds significant importance for gaining a deeper understanding of the pathological mechanisms underlying NMOSD and may pave the way for innovative treatments.

## Data Availability

The original contributions presented in the study are included in the article/supplementary material. Further inquiries can be directed to the corresponding author.
